# Partner support for women’s antidepressant treatment and its association with depressive symptoms in pregnant women, mothers, and women planning pregnancy

**DOI:** 10.1007/s00737-024-01435-3

**Published:** 2024-02-02

**Authors:** Tania Nasrin, Fatima Tauqeer, Ludvig D. Bjørndal, Sarah Kittel-Schneider, Angela Lupattelli

**Affiliations:** 1 Department of Community Medicine and Global Health, Faculty of Medicine, University of Oslo, 0316 Oslo, Norway; 2 PharmacoEpidemiology and Drug Safety Research Group, Department of Pharmacy, Faculty of Mathematics and Natural Sciences, University of Oslo, Blindern, PO Box 1068, 0316 Oslo, Norway; 3 PROMENTA Research Center, Department of Psychology, University of Oslo, 0317 Oslo, Norway; 4 Department of Psychiatry and Neurobehavioural Science, University College Cork, Acute Adult Mental Health Unit, Cork University Hospital, Wilton, Cork, T12DC4A Ireland

**Keywords:** Partner support, Antidepressant, Depressive symptoms, Pregnancy, Postpartum, Preconceptional

## Abstract

**Purpose:**

To examine the association between partner support for women’s antidepressant treatment and depressive symptoms in pregnant women, those planning pregnancy, and mothers who ever used antidepressants.

**Methods:**

We included 334 women (*n*=44 planners, *n*=182 pregnant, *n*=108 mothers) ever treated with antidepressants within the HEALTHx2 study, a web-based cross-sectional study conducted across Norway in June 2020 to June 2021. The Edinburgh Postnatal Depression Scale and two questions of the Patient Health Questionnaire measured depressive symptoms, by degree of severity and for depressed mood, anxiety, and anhedonia sub-dimensions. Partner support was measured using one item from the Antidepressant Compliance Questionnaire. Association was estimated via unadjusted and adjusted linear and logistic regression models.

**Results:**

Being unsupported by the partner was associated with increased odds of reporting moderate-to-very-severe depressive symptoms in mothers (adjusted odds ratio (aOR), 3.57; 95% confidence interval (CI), 1.04–12.19) and pregnant women (aOR, 3.26; 95% CI, 0.95–11.14), relative to being supported. Pregnant women (adjusted mean difference (β), 0.76; 95% CI, 0.14–1.38) and mothers (β, 0.93; 95% CI, 0.23–1.64) with no support for their antidepressant treatment presented greater symptoms of anhedonia; for women planning pregnancy, this association emerged in relation to anxiety symptoms (β among non-users of antidepressant, 2.58; 95% CI, 1.04–4.13).

**Conclusions:**

Partner support for women’s antidepressant treatment may play a key role in depressive symptoms severity and the subtypes of anhedonia and anxiety, among women planning pregnancy, pregnant women, and mothers. This highlights the importance of partner inclusion in the complex decision-making process for antidepressant treatment around the time of pregnancy.

## Introduction

Perinatal depression, which refers to depression that occurs between the beginning of pregnancy and the end of the first postpartum year, has an estimated prevalence of 10–15% and 25% in economically developed and developing countries, respectively (Woody et al. 2017). There is high comorbidity between depression and anxiety (Falah-Hassani et al. 2016, 2017). Anxiety, together with anhedonia, is a prominent and severe symptom dimension of depression with postpartum onset (Putnam et al. 2017), and anxious depression is often challenging to treat in clinical practice (Fava et al. 2008).

In moderate-to-severe cases of perinatal depression and/or anxiety, or if first-line psychotherapy has been ineffective, antidepressant medication, mainly selective serotonin reuptake inhibitors (SSRIs), is the preferred pharmacological choice. About 2–3% of women in Europe and 4–10% in North America receive SSRIs during pregnancy (Yonkers et al. 2009; Battle et al. 2013; Zoega et al. 2015).Pregnancy remains a major driver of antidepressant discontinuation (Trinh et al. 2022), and interruption of treatment may have negative implications for maternal perinatal mental health outcomes (Trinh et al. 2023).

Multiple sociodemographic, psychological, and health-related factors in women have been found to increase the risk of perinatal depression, including support from the partner (Vanwetswinkel et al. 2022). The quality of the woman’s relationship with her partner and interpersonal support is strongly associated with postpartum depression (Dennis and Ross 2006; Misri et al. 2013; Vanwetswinkel et al. 2022). When it comes to the complex decision-making about antidepressant treatment at the time around pregnancy, emotional support may reduce the woman’s decisional conflict about whether to use antidepressants (Walton et al. 2014). Examining the role of partner agreement with women’s antidepressant treatment could therefore be important in order to better understand the complex decision-making process surrounding pharmacological treatment of perinatal depression.

In a sample of pregnant women, mothers, and women planning pregnancy ever treated with antidepressants, we aimed to assess the association between partner support for their antidepressant treatment and women’s depressive symptoms, according to level of severity and for the subtypes of anhedonia, anxiety, non-specific depressive symptoms, and depressed mood. To understand any effect modification by current antidepressant treatment, we stratified the analyses by antidepressant use at the time of symptom reporting. Mental health was assessed in women planning pregnancy, pregnant, and mothers up to 5 years postpartum given its importance for the women and their offspring (Gjerde et al. 2017).

## Methods

### Study design and participants

**Fig. 1:**
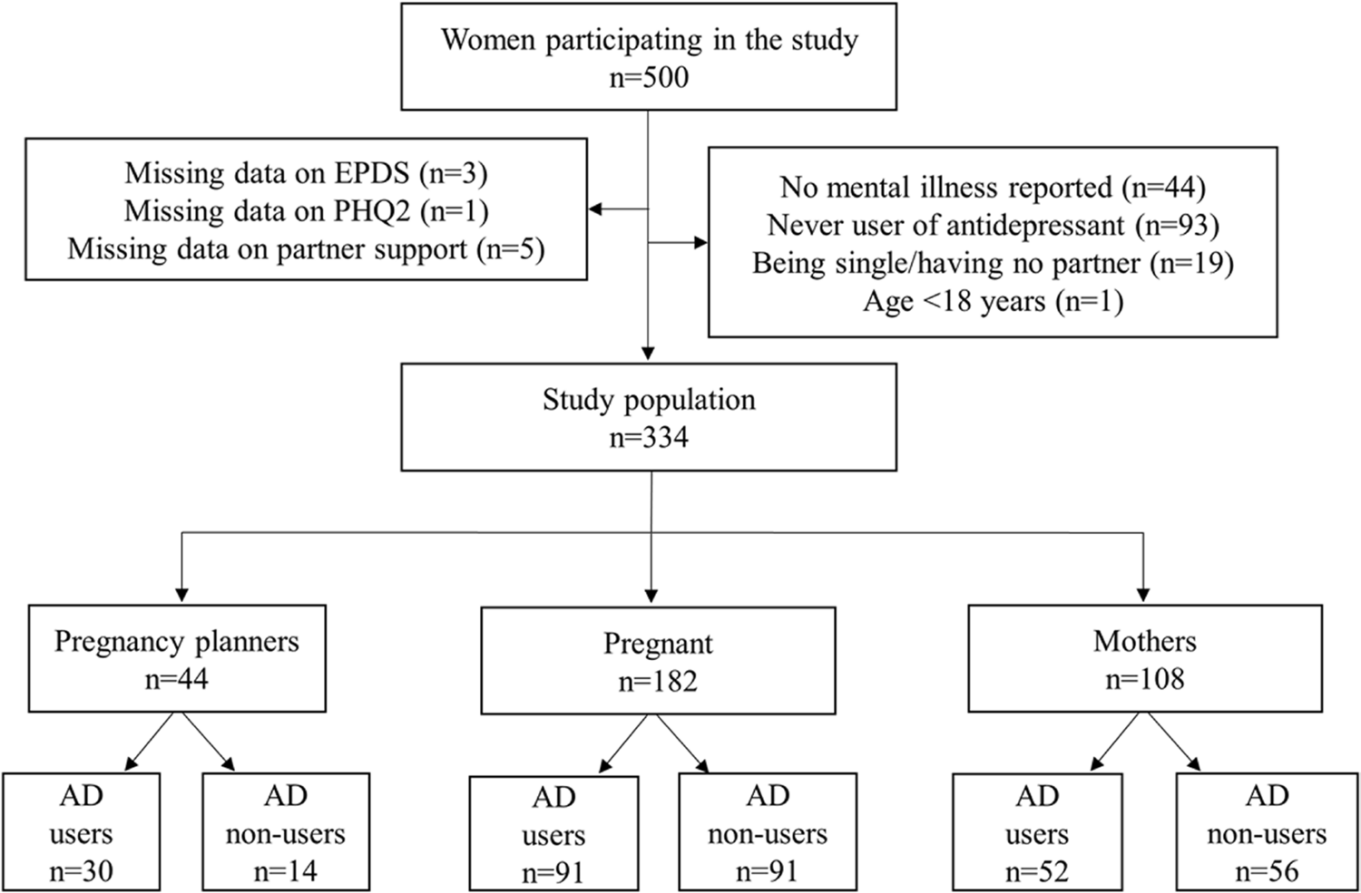
Data flow to achieve the final study population. Abbreviations: AD=Antidepressant; EPDS=Edinburgh Postpartum Depression Scale; PHQ2= two questions of the Patient Health Questionnaire

Participants were recruited from the HEALTHx2 study, a cross-sectional, sequential mixed-methods study, where Norwegian data were collected nationwide through between June 2020 and June 2021 using an electronic questionnaire. The questionnaire was administered via “Nettskjema” provided by the University of Oslo and was available to participants through pregnancy and motherhood-related websites, apps, social media, and brochures about the study distributed at various psychiatric outpatient and inpatient clinics, and maternal health clinics. The complete questionnaire and recruitment strategy have been published elsewhere (Bjørndal et al. 2022). Women could participate in the study anonymously or by using their national ID number. Women were considered for inclusion if they (i) were aged 18–55 years; (ii) were planning a pregnancy; were pregnant or had given birth within the last 5 years; and (iii) had a current or past mental illness and had been offered antidepressant treatment within the last 5 years. A pilot study was carried out in May 2020, which elicited no major changes to the questionnaire. In this study, we used only quantitative cross-sectional data and further restricted the sample to women with a partner at the time of study participation and who had ever used antidepressants (Fig. 1).

### Partner support

Partner support for the woman’s antidepressant treatment was measured using one item of the “Antidepressant Compliance Questionnaire” (ACQ) tool (Demyttenaere et al. 2004), which is “My partner agrees that antidepressants are a suitable treatment for my condition.” The ACQ assesses patients’ perceived doctor–patient relationship, preserved autonomy, positive beliefs about antidepressants, and partner agreement to treatment (Demyttenaere et al. 2004). Women could rate the extent to which they agreed with the one item on a 4‐point Likert scale with the response alternatives “mostly disagree,” “disagree,” “agree,” and “mostly agree.” We classified women as having partner support whenever they agreed/mostly agreed with the statement, and as having no partner support when they disagreed/mostly disagreed. The ACQ has been previously used in a perinatal population (Misri et al. 2013). The ACQ was translated to Norwegian and back-translated using two independent translators.

### Mental health outcomes

The main outcome variables were current (i) depressive symptoms, measured by the Edinburgh Depression Scale (EPDS) (Cox and Holden 2003; Cox et al. 1987), and (ii) symptoms of depressed mood and anhedonia, measured by two items of the Patient Health Questionnaire (PHQ-2) (Williams et al. 2002), as reported by pregnant women, mothers, and women planning pregnancy. The EPDS is the most widely used international screening questionnaire for symptoms of depression during pregnancy and postpartum and has demonstrated satisfactory Cronbach’s alpha reliability (0.87) (Cox et al. 1987; Cox and Holden 2003). The EPDS has been validated in a Norwegian sample (Eberhard-Gran et al. 2001). Responses to each of the 10 EPDS items scored on an ordinal scale from 0 to 3, resulting in a total score of 0–30 (Cox and Holden 2003; Cox et al. 1987). Higher scores indicate worse symptomatology in the last week. To better understand associations by symptom severity, we categorized the EPDS into the following severity levels, as done by prior research (Putnam et al. 2017): “no symptoms” (score <10), “mild-to-moderate” (10–16), and “moderate-to-very-severe” (17–30) depressive symptoms. We collapsed the “moderate to severe” and “very severe” into a single category due to low sample size. Because several studies have found the EPDS to be multidimensional (Tuohy and McVey 2008; Putnam et al. 2017), we also explored the three EPDS subtypes comprising “non-specific depressive symptoms” (items 7–10), “anxiety symptoms” (items 3–5), and “anhedonia” (items 1 and 2). Mean scores were calculated for the total EPDS and for each individual EPDS subtype score.

The PHQ-2 was administered to measure depressed mood and anhedonia over the past month, as these assess the two most relevant DSM-IV depression criteria (Association 1994; Williams et al. 2002). We defined women as having current symptoms of depressed mood and anhedonia (yes/no) if they answered positively to both questions. The PHQ-2 has shown adequate sensitivity (69–84%) and specificity (79–84%) in a pregnant population (Vlenterie et al. 2017).

### Health-related and sociodemographic factors

Participants self-reported their previous and current mental health within a predefined list including depression, anxiety, obsessive-compulsive disorders, eating disorders, and other mental illness, along with the periods they had the illness (Bjørndal et al. 2022). To measure women’s mental health burden, we counted the number of different illnesses reported across the available periods. Participants could report if they had previously received or were currently receiving psychological therapy (dichotomized as yes/no). Use and timing of antidepressant treatment were self-reported using a drop-down list containing all antidepressant medications marketed in Norway. The list contained the substance names of the individual antidepressants with examples of brand names to aid recall.

Perceived stigma related to mental illness was measured using four selected items from the “Attitudes Toward Seeking Professional Psychological Help Scale” (ATSPPHS) (Mackenzie et al. 2004). Participants could indicate the extent to which they agreed or disagreed on each item of the scale ranging from 0 to 4. Scores across items were summed (range 0–20), and a greater score corresponded to more indifference to stigma, i.e., more positive attitudes. The ATSPPHS was translated to Norwegian and back-translated using two independent translators.

The perceived doctor–patient relationship was measured via three ACQ items (Demyttenaere et al. 2004): “My doctor has explained properly about antidepressants, their action and side‐effects”; “My doctor shows sufficient consideration for my views and feelings about his treatment with antidepressants”; and “My doctor fully understands my condition.” Women could rate the extent to which they agreed with the statements on a 4‐point Likert scale, from “mostly disagree = 4” to “disagree = 3,” “agree = 2,” and “mostly agree = 1.” We calculated the sum score and divided it by the number of items (score range 1–4); thus, a greater score indicates a worse patient–doctor relationship.

Information on sociodemographic, i.e., age, educational attainment, and work situation, pregnancy and life-style characteristics, i.e., gravidity, planned pregnancy, smoking and alcohol consumption in pregnancy and postpartum, and body mass index (BMI), was collected via self-report using questions based on a prior web-based, cross-sectional study conducted among pregnant women in Norway (Lupattelli et al. 2014).

Based on a directed acyclic graph (DAG) (Textor et al. 2011) and subject knowledge, our assumed sufficient set of confounders included maternal age, education, occupation, psychological treatment, gravidity, number of reported mental illnesses, doctor support on antidepressant treatment, and perceived stigma.

### Data analysis

We first conducted descriptive statistics. We then fit crude and adjusted multiple general linear models with a robust standard error, to determine the association of partner support with the total and sub-dimension scores of the EPDS. To examine associations with the dichotomized outcome variables (that is, degree of severity on the EPDS, and depressed mood and anhedonia on the PHQ-2), we conducted crude and adjusted logistic regression. The adjusted linear and logistic models included our sufficient set of confounders and were conducted separately in planners, pregnant women, and mothers. We did not conduct logistic regression analysis among planners due to the low sample size. Because the current use of antidepressants at the time of reporting could be a consequence of the partner support rather than a preceding cause, we stratified our linear regression analysis by this factor and presented strata-specific point estimates. This stratification was not done for the logistic regression models due to low statistical power. Results are presented as crude and adjusted mean differences and odds ratios (ORs) with the corresponding 95% confidence intervals (CI).

Under the assumption that data were missing at random, we imputed incomplete data on the confounding variables via multiple imputation with chained equation (ten replications). The imputation model included baseline and health-related factors and auxiliary variables. All statistical analyses were conducted using STATA MP v. 17.

### Ethics

This study was carried out in compliance with the Helsinki Declaration. Electronic informed consent was given by each participant. The Regional Ethics Committee in Norway, region Southeast (reference number 94347), and the Norwegian Centre for Research Data (reference number 943055) approved the study.

## Results

Of the 753 women who indicated their willingness to participate in the study, 500 (66.4% response rate) consented. Figure 1 shows the data flow and exclusion criteria applied to achieve the final study population of 334 women who had a partner at the time of study participation. Of these, 44 were planners (13.2%), 182 were pregnant (54.5%), and 108 were mothers (32.3%). Of the planners, 50.0% reported no prior pregnancy, whereas for pregnant women and mothers, this proportion was 43.4% and 41.7%, respectively. Overall, 15.9% of pregnant women, 22.7% of planners, and 23.2% of mothers reported that they did not receive support from their partner. Women with no partner support had more often no prior pregnancy, greater use of other medications, perceived greater stigma toward their mental illness, and worse relationship with their doctor, relative to their counterpart (Table 1).

**Table 1:** Sociodemographic and health-related characteristics of the study sample (*N*=334)

	Women planning pregnancy (*n*=44)		Pregnant women (*n*=182)		Mothers (*n*=108)	
	Partner support (*n*=34)	No partner support (*n*=10)	Partner support (*n*=153)	No partner support (*n*=29)	Partner support (*n*=83)	No partner support (*n*=25)
	***n* (%)**					
Age (years)						
<25	<5	-	11 (7.2)	<5	<5	<5
25–35	29 (85.3)	7 (70.0)	117 (76.5)	26 (89.7)	61 (73.5)	20 (80.0)
>35	<5	<5	25 (16.3)	<5	17 (20.5)	<5
Educational attainment						
Low	<5	-	<5	<5	<5	<5
Medium	7 (20.6)	5 (50.0)	34 (22.2)	5 (17.2)	21 (25.3)	<5
High	26 (76.5)	5 (50.0)	116 (75.8)	22 (75.9)	59 (71.1)	20 (80.0)
Work situation						
Student	<5	-	10 (6.5)	-	7 (8.4)	<5
Homemaker	<5	-	9 (5.9)	<5	6 (7.2)	-
Health worker	<5	<5	27 (17.6)	7 (24.1)	13 (15.7)	7 (28.0)
Other paid work	20 (58.8)	7 (70.0)	90 (58.8)	17 (58.6)	47 (56.6)	10 (40.0)
Job seeker	<5	-	5 (3.3)	-	<5	<5
Disabled/social support	<5	<5	12 (7.8)	<5	7 (8.4)	<5
Gravidity^a^						
No prior pregnancy	15 (44.1)	7 (70.0)	65 (42.5)	14 (48.3)	33 (39.8)	12 (48.0)
Yes, prior pregnancy	19 (55.9)	<5	88 (57.5)	15 (51.7)	50 (60.2)	13 (52.0)
Planned pregnancy						
Yes	NA	NA	115 (75.2)	20 (69.0)	55 (66.3)	16 (64.0)
Not planned/not completely unexpected	NA	NA	38 (24.8)	9 (31.0)	28 (33.7)	9 (36.0)
Smoking^b^						
No	32 (94.1)	8 (80.0)	145 (94.8)	28 (96.6)	78 (94.0)	25 (100)
Yes, sometimes/daily	<5	<5	8 (5.3)	<5	5 (6.0%)	-
Alcohol consumption in pregnancy^c^						
No	NA	NA	148 (96.7)	29 (100)	79 (95.2)	25 (100)
Yes	NA	NA	5 (3.3)	-	<5	-
Psychological treatment						
Yes	15 (44.1)	7 (70.0)	68 (44.4)	14 (48.3)	50 (60.2)	15 (60.0)
No	19 (55.9)	<5	81 (52.9)	14 (48.3)	30 (36.1)	10 (40.0)
Missing	-	-	<5	<5	<5	-
Antidepressant use at the time of study participation						
Yes	25 (73.5)	5 (50.0)	79 (51.6)	12 (41.4)	35 (42.2)	17 (68.0)
No	9 (26.5)	5 (50.0)	74 (48.4)	17 (58.6)	48 (57.8)	8 (32.0)
Other medication use in the period 6 months before pregnancy^d^						
Analgesics and painkillers (yes)	NA	NA	25 (16.3)	10 (34.5)	17 (20.5)	8 (32.0)
Other psychotropics (yes)	NA	NA	23 (15.0)	13 (44.8)	17 (20.5)	5 (20.0)
	**Mean** (**SD)**					
Perceived patient–doctor relationship	1.8 (0.7)	2.4 (1.0)	2.0 (0.7)	2.2 (0.8)	2.1 (0.8)	2.6 (0.9)
Perceived stigma for mental illness	9.9 (3.3)	9.0 (3.9)	9.9 (4.2)	8.0 (3.9)	8.1 (3.9)	6.6 (4.8)
Self-reported number of mental illnesses	2.4 (1.1)	3.2 (1.1)	2.2 (1.0)	2.6 (1.2)	2.4 (0.9)	2.5 (1.2)
BMI^e^	26.7 (5.3)	24.0 (3.9)	26.0 (4.9)	26.1 (6.6)	25.9 (5.9)	25.5 (5.6)

Abbreviations: *BMI* body mass index

Missing values on doctor support and perceived stigma were in total observed in less than 5 observations

^a^Includes also pregnancies ending in spontaneous or elective abortions or stillbirths

^b^Smoking status was measured at the time of reporting for women planning pregnancy, and during pregnancy for pregnant and mothers

^c^Alcohol consumption was measured only in pregnant women and mothers, and relates to use during gestation after awareness of the pregnancy

^d^Use of other medications was not measured among pregnancy planners

^e^BMI was measured as current BMI for planners and BMI before pregnancy for pregnant women and mothers

### Partner support and depressive symptoms

Women with no partner support reported higher depressive symptoms as measured by the total EPDS score, than women having support (Table 2). For women planning pregnancy and pregnant women, the greatest absolute difference in symptoms severity by partner support status was observed for the non-specific depressive symptoms subtype. The proportions of women having moderate-to-very-severe depressive symptoms based on the EDPS, or depressed mood and anhedonia as measured by the PHQ-2, were consistently higher in women having no partner support than their comparators (Table 2).

**Table 2:** Depressive symptoms in women planning pregnancy, pregnant women, and mothers as measured by the main and sub-dimensions of the EPDS and the PHQ-2 scale, according to partner support for the woman’s antidepressant treatment

	Women planning pregnancy (*n*=44)		Pregnant women (*n*=182)		Mothers (*n*=108)	
	Partner support (*n*=34)	No partner support (*n*=10)	Partner support (*n*=153)	No partner support (*n*=29)	Partner support (*n*=83)	No partner support (*n*=25)
Depressive symptoms	**Mean (95% CI)**					
EPDS sum score ^a^	11.2 (9.6, 12.7)	14.9 (11.2, 18.6)	9.2 (8.4, 10.0)	12.3 (10.3, 14.3)	9.7 (8.5, 10.8)	13.0 (10.5, 15.5)
EPDS sub-dimensions						
Anhedonia	1.2 (0.8, 1.7)	1.9 (0.9, 2.9)	1.0 (0.8, 1.2)	1.8 (1.3, 2.4)	1.0 (0.8, 1.3)	2.2 (1.5, 2.9)
Anxiety symptoms	5.0 (4.3, 5.8)	5.9 (4.7, 7.0)	4.2 (3.8, 4.6)	5.0 (4.3, 5.7)	4.4 (3.9, 4.9)	5.0 (4.1, 5.9)
Non-specific depressive symptoms	3.4 (2.7, 4.1)	5.1 (3.1, 7.1)	2.5 (2.2, 2.9)	3.7 (2.7, 4.6)	2.8 (2.3, 3.3)	4.2 (3.2, 5.3)
	**Proportion percent (95% CI)**					
Mild-to-moderate depressive symptoms (EPDS score 10–16)	58.8 (53.6, 63.8)	60.0 (50.2, 69.2)	41.2 (38.8, 43.5)	48.3 (42.6, 53.9)	37.3 (34.2, 40.5)	44.0 (38.0, 50.1)
Moderate-to-very-severe depressive symptoms (EPDS score 17–30)	8.8 (6.1, 12.2)	20.0 (12.9, 28.6)	4.6 (3.6, 5.6)	17.2 (13.2, 21.8)	13.3 (11.1, 15.6)	20.0 (15.4, 25.2)
Symptoms of depressed mood and anhedonia (PHQ-2)	61.7 (43.6, 77.8)	80.0 (44.3, 97.5)	35.3 (27.7, 43.4)	62.1 (42.2, 79.3)	31.3 (21.6, 42.4)	60.0 (38.7, 78.9)

Abbreviations: *EPDS* Edinburgh Postnatal Depression Scale; *PHQ2* Patient Health Questionnaire-2

^a^The EPDS total score has range 0–30, with increasing score indicating greater depressive symptoms

^b^Anhedonia (EPDS items 1–2) has range 0–6; anxiety symptoms (EPDS items 3–5) has range 0–9; non-specific depressive symptoms (EPDS items 7–10) has range 0–12. Greater score indicates greater symptoms severity on the measured subscale

In the adjusted regression models (Table 3), pregnant women who were unsupported by their partner reported greater depressive symptoms on the total EPDS (adjusted mean difference (β)=2.49; 95% CI, 0.39–4.58) than those being supported. This association emerged also among planners and mothers currently taking antidepressants. There was an association between having no partner support and greater symptoms of anhedonia in both pregnant women (adjusted β=0.76; 95% CI, 0.14–1.38) and mothers (adjusted β, 0.93; 95% CI, 0.23–1.64), but for the latter group, the positive association was only evident among antidepressant users. Only among pregnancy planners, being unsupported was associated with greater anxiety symptoms.

**Table 3:** Association of depressive symptoms in women planning pregnancy, pregnant women, and mothers as measured by the main and sub-dimensions of the EPDS with partner support, overall, and stratified by current antidepressant treatment in the woman

	Women planning pregnancy		Pregnant women		Mothers	
	Crude model	Adjusted model^a^	Crude model	Adjusted model^a^	Crude model	Adjusted model^a^
	β (95% CI)	β (95% CI)	β (95% CI)	β (95% CI)	β (95% CI)	β (95% CI)
	**EPDS, total score**					
No partner support vs support	3.72 (0.27, 7.17)^‡^	1.97 (−0.14, 4.07)	3.08 (1.03, 5.12)^‡^	2.49 (0.39, 4.58)^‡^	3.35 (0.79, 5.91)^‡^	2.34 (−0.32, 5.00)
Users of antidepressants	5.36 (0.21, 10.51)^‡^	3.24 (0.51, 5.97)^‡^	3.45 (0.34, 6.56)^‡^	1.84 (−1.34, 5.03)	4.68 (1.17, 8.18)^‡^	3.37 (0.25, 6.50)^‡^
Non-users of antidepressants	3.62 (−0.00, 7.25)	0.04 (−4.60, 4.68)	2.80 (0.06, 5.54)^‡^	2.04 (−0.70, 4.79)	1.40 (−1.86, 4.65)	−2.01 (−6.31, 2.28)
	***EPDS, sub-dimensions***					
	**Anhedonia**					
No partner support vs support	0.66 (−0.26, 1.59)	0.20 (−0.49, 0.90)	0.79 (0.21,1.37)^‡^	0.76 (0.14, 1.38)^‡^	1.18 (0.48, 1.87)^†^	0.93 (0.23,1.64)^‡^
Users of antidepressants	1.36 (0.04, 2.68)^‡^	0.37 (−0.84, 1.58)	0.65 (−0.17, 1.47)	0.38 (−0.46, 1.21)	1.64 (0.73, 2.55)^†^	1.20 (0.36, 2.04)^‡^
Non-users of antidepressants	−0.02 (−1.05, 1.00)	−1.12 (−3.14, 0.89)	0.91 (0.10, 1.72)^‡^	0.87 (−0.01,1.75)	0.46 (−0.46, 1.38)	−0.39 (−1.41, 0.63)
	**Anxiety symptoms**					
No partner support vs support	0.87 (−0.31, 2.05)	0.68 (−0.80, 2.15)	0.80 (0.03,1.57)^‡^	0.58 (−0.19, 1.35)	0.62 (−0.32, 1.56)	0.07 (−0.92, 1.06)
Users of antidepressants	0.84 (−0.24, 1.92)	1.18 (0.28, 2.09)^‡^	1.06 (−0.17, 2.30)	0.71 (−0.52, 1.94)	1.06 (−0.20, 2.33)	0.44 (−0.76, 1.65)
Non-users of antidepressants	2.20 (0.11, 4.29)^‡^	2.58 (1.04, 4.13)^†^	0.67 (−0.31, 1.64)	0.49 (−0.46, 1.44)	0.02 (−1.26, 1.30)	−1.25 (−2.67, 0.18)
	**Non-specific depressive symptoms**					
No partner support vs support	1.72 (−0.07, 3.50)	0.87 (−0.02, 1.75)	1.13 (0.17, 2.08)^‡^	0.85 (−0.13, 1.82)	1.47 (0.37, 2.57)^‡^	1.22 (0.05, 2.38)^‡^
Users of antidepressants	2.76 (−0.09, 5.61)	1.48 (−0.10, 3.07)	1.39 (−0.12, 2.89)	0.63 (−1.02, 2.28)	1.78 (0.31, 3.26)^‡^	1.34 (0.05, 2.63)^‡^
Non-users of antidepressants	0.78 (−1.06, 2.61)	−0.81 (−3.77, 2.14)	0.85 (−0.41, 2.11)	0.44 (−0.88, 1.75)	1.00 (−0.56, 2.56)	−0.20 (−2.38, 1.99)

^a^Adjusted for maternal age, education, occupation, recent receipt of psychological treatment, gravidity, number of reported psychiatric illnesses, doctor support for antidepressant treatment, and stigma. The models are additionally stratified by antidepressant use status at the time of study participation

^†^Indicates* p*-value ≤0.001; ^‡^indicates *p*-value <0.05

Upon dichotomization of the outcome measures (Table 4), being unsupported by the partner was found to be associated with increased odds of reporting moderate-to-very-severe depressive symptoms by mothers (adjusted OR, 3.57; 95% CI, 1.04–12.19) and pregnant women (adjusted OR, 3.26; 95% CI, 0.95–11.14), relative to having partner support. This association emerged also for symptoms of depressed mood and anhedonia in both groups (Table 4).

**Table 4:** Association of depressive symptoms severity and depressed mood/anhedonia in pregnant women and mothers, as measured by the EPDS and the PHQ-2, with partner support ^a^

	Pregnant women		Mothers	
No partner support vs support	Crude OR (95% CI)	Adjusted OR (95% CI)^b^	Crude OR (95% CI)	Adjusted OR (95% CI)^b^
Mild-to-moderate depressive symptoms (EPDS score 10-16)	1.95 (0.79, 4.81)	1.49 (0.53, 4.17)	1.77 (0.63, 4.97)	1.29 (0.33, 5.01)
Moderate-to-very-severe depressive symptoms (EPDS score 17–30)	5.06 (1.97, 12.98)^†^	3.26 (0.96, 11.14)	2.88 (1.07, 7.81)^‡^	3.57 (1.04, 12.19)^‡^
Symptoms of depressed mood and anhedonia (PHQ-2)	3.00 (1.32, 6.83)^‡^	2.68 (1.09, 6.60)^‡^	3.29 (1.30, 8.33)^‡^	3.71 (1.22, 11.32)^‡^

^a^Association results for women planning pregnancy are not presented due to low statistical power

^b^Adjusted for maternal age, education, occupation, recent receipt of psychological treatment, gravidity, number of reported psychiatric illnesses, doctor support for antidepressant treatment, and stigma. The adjusted models could not be stratified by antidepressant use status at the time of study participation due to low statistical power

^†^Indicates *p*-value ≤0.001; ^‡^indicates *p*-value <0.05

## Discussion

This study reports novel knowledge on the association between partner support for the woman’s antidepressant treatment and self-reported depressive symptoms in a sample of pregnant women, mothers, and women planning pregnancy ever treated with antidepressants. In our sample, approximately one in five women reported being unsupported by their partner. This is somewhat encouraging as it shows that the majority of women receive partner support for their antidepressant treatment. Nevertheless, lack of partner support was consistently associated with greater and clinically relevant depressive symptoms, in women planning pregnancy, pregnant women, and mothers. Several of our findings have important implications for clinical practice and family support strategies and highlight the importance of partners in the counseling and decision-making process for antidepressant treatment at the time around pregnancy and childbirth.

We found that pregnant women and mothers who lacked partner support had a substantially increased likelihood of displaying clinically relevant symptoms of depression, that is, an EDPS score >12–13 (Cox et al. 1987), including depressed mood and anhedonia, compared to those who had such support. This finding is indicative of the important role of partner support for perinatal mental health in women. Although the absence of sufficient comparable data poses challenges for comparing our results to earlier studies, our results are consistent with some prior research on the negative impact of poor partner relationships on women’s depressive symptoms (Gremigni et al. 2011; Stapleton et al. 2012; Davey-Rothwell et al. 2017).

One key finding is that lack of partner support is associated with different clinical subtypes of depressive symptoms in women planning pregnancy, pregnant, or mothers. Identifying women with different depressive symptom subtypes is critical at the phase of prognosis and for tailoring treatment strategies to individual women’s needs (Putnam et al. 2015), as each subtype is characterized by different degrees of severity and response to treatment (Fava et al. 2008). In our study, mothers and pregnant women with no partner support reported greater symptoms of anhedonia than their counterparts did, but in mothers, this association emerged only among those who were currently using antidepressants. Anhedonia has been found to be a prominent and severe symptom dimension with postpartum onset (Putnam et al. 2017), which may persist despite antidepressant treatment. This results could be explained by information bias and confounding by disease severity, as women with more severe symptoms and thereby on antidepressants may perceive the support of their partner differently from women with lower symptom severity. Another possible explanation is that lack of partner support may lead to decreased antidepressant adherence by the woman in the context of ongoing treatment.

Pregnancy planners with no partner support presented greater anxiety symptoms than women having such support, although the mean difference was of greater magnitude among non-users of antidepressants than among users. Women planning pregnancy constitute an understudied population group (Barker et al. 2020), and most research to date has focused on depression during and after pregnancy (Tuohy and McVey 2008; Putnam et al. 2017). Women being unsupported by their partner may discontinue their antidepressant treatment also at the phase of preconception, thereby entering the pregnancy with clinically relevant symptoms of anxiety, which may increase the risk of multiple negative outcomes for both mother and child (Trinh et al. 2022). Future studies of larger sample size are needed to better understand the role of partner support on the mental health of women planning pregnancy.

Support from partners, family, friends, and professionals can contribute to improving the mental health of pregnant women, including symptoms of depression and anxiety (Bernazzani et al. 2004; Lundsberg et al. 2020). Most studies have investigated partner support in terms of providing companionship, doing things to help, and feeling included (Dennis and Ross 2006), but the partner agreement with the woman’s drug treatment should be deemed equally important. When it comes to the complex decision-making surrounding antidepressant treatment, it is crucial that the partner, together with and for the woman, develops an evidence-based understanding of the benefits and possible risks of antidepressant treatment at the phase of preconception through pregnancy and late postpartum, to facilitate the decision-making process and support the woman’s mental health. This could be a byproduct of doctor–patient relationship, which yields the importance of education for couples but also patient-provider dyads.

## Strengths and limitations

One major strength of our study is that depressive outcomes were measured using two validated screening tools, EPDS (Cox et al. 1987; Cox and Holden 2003) and PHQ-2 (Williams et al. 2002); the EPDS is specifically tailored to the perinatal population, and the PHQ-2 has been used in pregnancy settings previously (Vlenterie et al. 2017). We studied depressive outcomes in terms of symptoms subtypes and across different times. The study had a considerable study size given the difficult-to-reach population. Several recruitment strategies were implemented to minimize the risk of selection bias. We collected a vast array of factors, including the use of antidepressants at the time of symptom reporting, perceived stigma toward mental illness, and patient–doctor relationship. We also conducted multiple imputations for missing data.

Our study has limitations that need to be considered when interpreting the results. The sample size for women planning a pregnancy was low, and women with no current partner were excluded from the analysis. Partner support was measured using one single item, which was also not related to a specific time point. This may have affected the granularity of this exposure in relation to the pregnancy status. Further, we did not ask women whether their decision to use or not use antidepressants was a consequence of their partner’s support or of the relationship with their doctors. The mental illnesses were self-reported by the participants and therefore dependent on the accuracy of the woman’s reporting. Women could retrospectively report on their past mental illness, which is dependent on recall and their current mental health status at the time of study participation. As the eligibility criteria included being offered antidepressant treatment in the last 5 years, primarily moderate-to-severe mental illness cases were targeted. We included women with different mental illnesses, for which the indicated treatment may vary in terms of dose and length, and this may have affected our results. One key limitation is the cross-sectional design of the study and lack of the temporal component. We cannot exclude the possibility of information bias, as women with more severe depressive symptoms may perceive the support of their partners differently from women with less severe symptoms. The use of an electronic questionnaire and multiple recruitment strategies did not permit the calculation of a conventional response rate, and bias due to self-selection cannot be ruled out. However, among the women expressing their willingness to participate or not in the study, the response rate was satisfactory (66%). The validity of web-based recruitment methods is now well-acknowledged (van Gelder et al. 2010, 2018), and the Internet penetration rate is almost 100% in women of childbearing age in Norway. We did not adjust our association analyses for current antidepressant use but rather stratified by this factor, as antidepressant use may be a consequence of partner support. Finally, we cannot exclude the possibility that the women who decided to participate in the study differed from the general birthing population of women with mental illnesses in ways that our analysis could not control for.

## Conclusion

Women planning pregnancy, pregnant women, and mothers being unsupported by their partner in relation to antidepressant treatment reported consistently greater and clinically relevant depressive symptoms when compared to women with such support. Lack of partner support is associated with different subtypes of depressive symptoms at different time points. In mothers and pregnant women, this association was prominent in relation to anhedonia and non-specific depressive symptoms, while among planners, for the anxiety dimension. Partners support likely plays an important role in the complex decision-making process regarding antidepressant treatment around the time of pregnancy and childbirth.

## Data Availability

All data relevant to the study are included in the article or uploaded as supplementary information. Researchers can apply for data access for subprojects within the overall aims of the main study “HEALTHx2.”
